# Phenotypic responses to light, water, and nutrient conditions in the allopolyploid *Arabidopsis suecica* and its parent species *A. thaliana* and *A. arenosa*: Does the allopolyploid outrange its parents?

**DOI:** 10.1002/ece3.8915

**Published:** 2022-05-13

**Authors:** Torbjørn Kornstad, Mikael Ohlson, Siri Fjellheim

**Affiliations:** ^1^ 365639 Department of energy and environment Norconsult AS Sandvika Norway; ^2^ 56625 Faculty of environmental sciences and natural resource management Norwegian University of Life Sciences Ås Norway; ^3^ 56625 Faculty of Biosciences Norwegian University of Life Sciences Ås Norway

**Keywords:** *Arabidopsis arenosa*, *Arabidopsis suecica*, *Arabidopsis thaliana*, fitness, genetic variation, phenotypic plasticity, polyploidy

## Abstract

Polyploid species possess more than two sets of chromosomes and may show high gene redundancy, hybrid vigor, and masking of deleterious alleles compared to their parent species. Following this, it is hypothesized that this makes them better at adapting to novel environments than their parent species, possibly due to phenotypic plasticity. The allopolyploid *Arabidopsis suecica* and its parent species *A*. *arenosa* and *A*. *thaliana* were chosen as a model system to investigate relationships between phenotypic plasticity, fitness, and genetic variation. Particularly, we test if *A*. *suecica* is more plastic, show higher genetic diversity, and/or have higher fitness than its parent species. Wild Norwegian populations of each species were analyzed for phenotypic responses to differences in availability of nutrient, water, and light, while genetic diversity was assessed through analysis of AFLP markers. *Arabidopsis arenosa* showed a higher level of phenotypic plasticity and higher levels of genetic diversity than the two other species, probably related to its outbreeding reproduction strategy. Furthermore, a general positive relationship between genetic diversity and phenotypic plasticity was found. Low genetic diversity was found in the inbreeding *A*. *thaliana*. Geographic spacing of populations might explain the clear genetic structure in *A*. *arenosa*, while the lack of structure in *A*. *suecica* could be due to coherent populations. Fitness measured as allocation of resources to reproduction, pointed toward *A*. *arenosa* having lower fitness under poor environmental conditions. *Arabidopsis suecica*, on the other hand, showed tendencies toward keeping up fitness under different environmental conditions.

## Graphical abstract

Does polyploid species outrange their parents in phenotypic response range, genetic diversity, and fitness? We investigated the allopolyploid Arabidopsis suecica and its diploid parents A. thaliana and A. arenosa and found no indications of this.

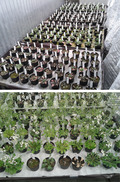

## 1 INTRODUCTION

Polyploidization is recognized as a driving force for angiosperm diversification and speciation (Wendel, 2015). Polyploid species possess more than two sets of chromosomes, acquired either by intraspecific genome doubling (autopolyploidy) or by merging of genomes of different species through hybridization (allopolyploidy). A newly formed polyploid combines genes from two individuals, and this opens for hybrid vigor and masking of deleterious, recessive alleles (te Beest et al., 2012). Neo‐ or subfunctionalization may lead to genetic innovation (Hegarty & Hiscock, 2008; Lynch & Force, 2000), and a high gene redundancy suggests that polyploids could withstand inbreeding and population bottlenecks better than their diploid counterparts (Song et al., 2012; te Beest et al., 2012). Following this, polyploids may harbor high levels of genetic diversity, especially if there are multiple origins of the polyploid species. The genomic changes and increased genetic diversity may lead to altered morphology, physiology, and ecology (Parisod et al., 2010). Generation of new expressional patterns and novel epigenetic variation could also contribute to this (Chen, 2007; Comai, 2005). Following this, polyploids could have an adaptive advantage in new or changing environments, giving the polyploid hybrid species a higher fitness than either of the parental species. The effects are expected to be more pronounced for allopolyploid species. At the same time, there are genetic forces associated with polyploidization that may be detrimental. For example, polyploidization is a process that changes the genome abruptly in just one generation, and this may lead to a notoriously unstable genome. A result may be unstable mitosis and meiosis, giving aneuploid cells, and problems with gene expression due to development of uneven relationships between genes and regulatory factors (Comai, 2005). Epigenetic re‐modeling could also cause instability in newly formed polyploids (Comai et al., 2003). Furthermore, polyploids often go through severe bottlenecks in their origins (Layman & Busch, 2018), which may reduce the level of genetic diversity. Even though polyploidization may bring both advantages and disadvantages, the view that polyploidization generates wider ecological and phenotypical variation and thus enables species to adapt quickly is widely accepted (Comai, 2005; Flagel & Wendel, 2009; Otto, 2007; te Beest et al., 2012). There are, however, also opposing views (Arrigo & Barker, 2012; Kellogg, 2016; Mayrose et al., 2011; Meyers & Levin, 2006).

The physiological effects of polyploidization are relatively little explored, but Soltis et al. (2016) pinpoint cases that are relatively well described: genome doubling within each cell lead to larger cells, again leading to larger stomata and vascular cells, higher photosynthetic rate and gas exchange due to the larger stomata, and increased susceptibility to drought due to larger xylem vessels, leading to differences in stress resistance between polyploids and their parents (Soltis et al., 2016). Furthermore, it is assumed that higher genetic diversity constitutes a foundation for higher fitness, hence if a polyploid species has a higher level of genetic diversity than its parent species this may be advantageous (Reed & Frankham, 2003).

The adaptive advantage of an allopolyploid may result from phenotypic plasticity and/or fitness homeostasis (Godfree et al., 2017; Scheiner, 1993; Stevens et al., 2020). Phenotypic plasticity is the ability to exhibit a wide range of phenotypes across varying environmental conditions (Bradshaw, 1965; Schlichting, 1986). However, this may not necessarily imply higher fitness. Fitness homeostasis is the ability to keep fitness as equal as possible between varying environmental conditions (Hulme, 2008; Richards et al., 2006). It is proposed that high phenotypic plasticity provides wider possibilities to adapt to new environments (Davidson et al., 2011; Sultan, 2000), while high fitness homeostasis could imply better abilities at coping with and adapting to stressful environments (Godfree et al., 2017; Hulme, 2008; Richards et al., 2006; Stevens et al., 2020). Summed up, a theoretical framework for a possible positive relationship between polyploidy and abilities to adapt is established (Flagel & Wendel, 2009). Nevertheless, conclusive results for polyploid species outcompeting their parent species in their ecological niche or expansion to niches unavailable to the parent species have been difficult to establish (Soltis et al., 2016). Note that in this work, we use phenotypic plasticity on species and population level, whereas phenotypic plasticity in its most strict sense refers to the ability of a single genotype to respond differently to various environments.

To investigate physiological effects of polyploidization and its possible role in giving adaptive advantages compared to parent species, we use the hybrid complex consisting of the allopolyploid species *Arabidopsis suecica* (Fr.) Norrl. ex O.E. Schulz and its two parent species, *A*. *thaliana* (L.) Heynh. and *A*. *arenosa* (L.) Lawalrée. *Arabidopsis suecica* originates from a hybridization between the mostly diploid *A*. *thaliana* and the mostly autotetraploid *A*. *arenosa* (Jakobsson et al., 2006; O'Kane et al., 1996), possibly within the eastern parts of *A*. *thaliana's* native range (Beck et al., 2008; Novikova et al., 2017). The formation of the species probably occurred through the fertilization of a female, unreduced *A*. *thaliana* gamete with a normal, male *A*. *arenosa* gamete from a tetraploid *A*. *arenosa* (Jakobsson et al., 2006; Novikova et al., 2018; Säll et al., 2003). It is believed to have originated between 12,000 and 300,000 years ago, somewhere south of its present native distribution in Sweden and Finland (Jakobsson et al., 2006; Novikova et al., 2017; Säll et al., 2003). Specifically, Novikova et al. (2017) suggest multiple origins, likely after the last glaciation maximum in Eastern Europe or central Eurasia. Burns et al. (2021) conclude that the process leading to the species *A*. *suecica* has been gradual, and they find no evidence of genome shock.

All three species are winter annuals, forming an overwintering basal rosette of leaves in the autumn and a flowering stem in the following spring (Baskin & Baskin, 1983). While *A*. *arenosa* is a strictly outcrossing species, *A*. *thaliana* and *A*. *suecica* are self‐fertilizing species, which set seeds regardless of whether they are pollinated or not (Säll et al., 2004). All three species prefer dry habitats. In Norway, *A*. *thaliana* often grows in dry meadows, rock crevices and on ledges, while the other two typically are found on sandy soils—often close to road verges and along railways (Elven, 2005).

In this paper, we investigate variation in phenotypic variables in response to different nutrient, light, and water treatments as well as genetic diversity in the diploid *A*. *thaliana* and *A*. *arenosa* and its polyploid daughter species *A*. *suecica*. Specifically, we ask the following research questions:

1. Does the allopolyploid *A*. *suecica* show more genetic diversity and larger phenotypic plasticity and/or fitness homeostasis than its parent species?
2. Are there any relationships between genetic diversity, fitness homeostasis, and phenotypic plasticity in the study species?

## 2 MATERIALS AND METHODS

### 2.1 Study area

**FIGURE 1:**
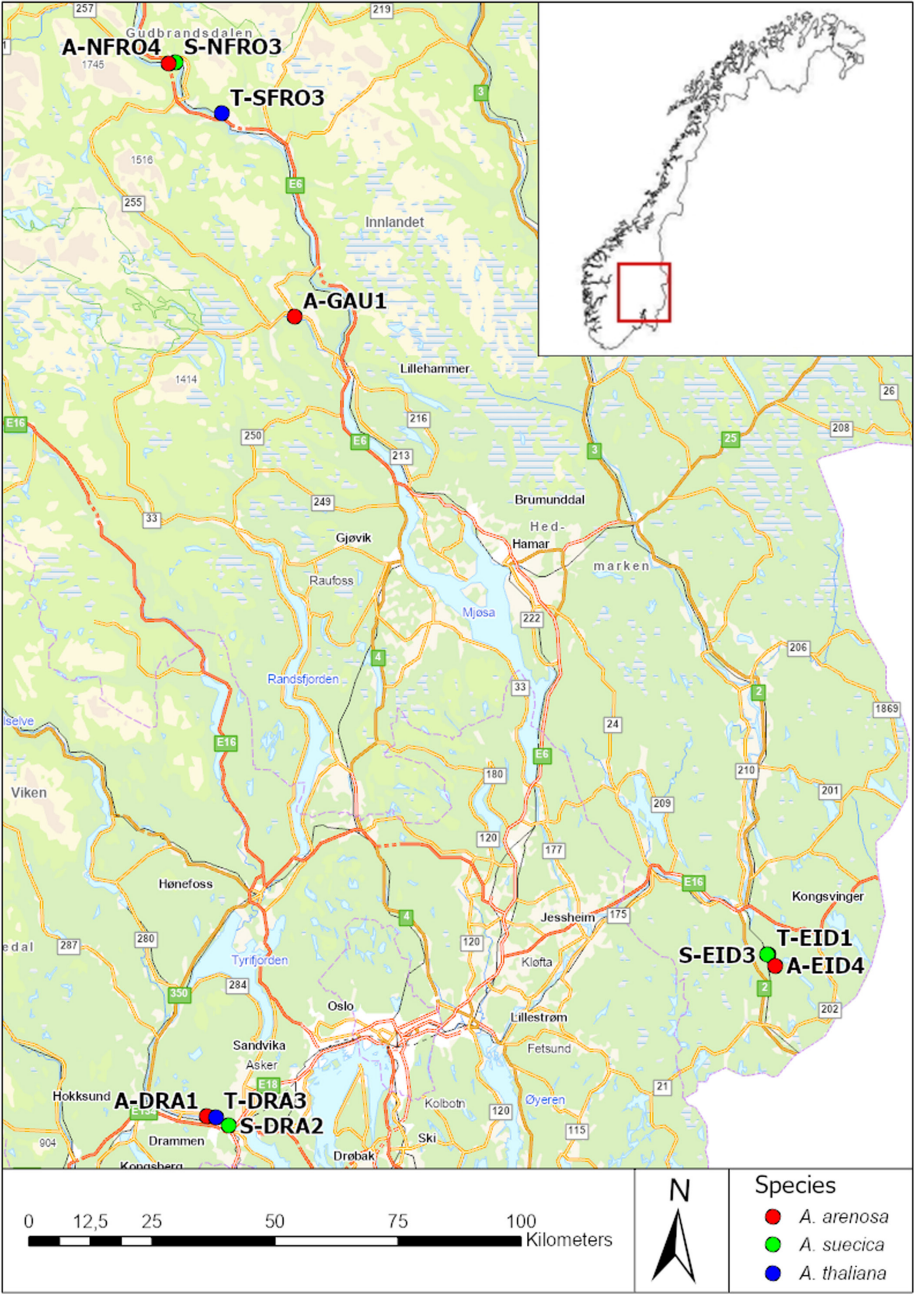
Map showing localities of populations where seeds were sampled for the experiment. Background map: Geodata AS

Seeds of *A*. *thaliana*, *A*. *suecica*, and *A*. *arenosa* were sampled from 10 wild populations in three different geographic areas in SE Norway (Figure 1). The number of sampled populations per species was three for *A*. *thaliana* and *A*. *suecica*, and four for *A*. *arenosa*. All species were sampled in each geographical area (Table 1).

**TABLE 1:** List of populations where seeds were sampled, specifying locality codes, locality names, what geographical areas the different localities belong to, species, collection date, latitude in degrees north (Lat (°N)), and longitude in degrees east (Long (°E))

Code	Locality name	Geographical area	Species	Collection date	Lat (°N)	Long (°E)
T‐EID1	Bakkeberget	Eidskog	*A. thaliana*	11.06.2012	60.111	12.123
S‐EID3	Åbogen stasjon	Eidskog	*A. suecica*	11.06.2012	60.109	12.116
A‐EID4	Pramhus	Eidskog	*A. arenosa*	11.06.2012	60.090	12.149
A‐DRA1	Berskog	Drammen	*A. arenosa*	21.06.2012	59.755	10.120
S‐DRA2	Drammen stasjon	Drammen	*A. suecica*	17.06.2012	59.741	10.202
T‐DRA3	Åslyveien	Drammen	*A. thaliana*	21.06.2012	59.756	10.154
T‐SFRO3	Kjorstad	Gudbrandsdal	*A. thaliana*	05.07.2012	61.579	9.894
S‐NFRO3	Kvam stasjon	Gudbrandsdal	*A. suecica*	05.07.2012	61.665	9.702
A‐NFRO4	Nymoen	Gudbrandsdal	*A. arenosa*	05.07.2012	61.663	9.676
A‐GAU1	Steinslia	Gudbrandsdal	*A. arenosa*	07.07.2012	61.220	10.228

For each population, 20 randomly chosen individuals were sampled. The life stage of the collected plants was not standardized. If a population consisted of less than 20 individuals, as many individuals as possible were sampled. The lowest number of individuals sampled per population was 6. The plants were dried, and the seeds extracted and transferred to 2‐ml tubes (Eppendorf, Hamburg, Germany).

### 2.2 Measurements of ploidy level and chromosomal numbers

In order to ensure that all populations of the study species had the expected chromosomal numbers and ploidy levels, DNA content was measured with flow cytometry. Seeds from each of the populations grown in the experiment were sown in pots and grown to a size where harvesting was permissible. For each population, three individuals were selected for harvesting. Leaves corresponding to a total area of 1–2 cm^2^ were harvested. Leaf harvesting was not standardized. Flow cytometry was performed, and DNA ratios were obtained by G. Geenen, Plant Cytometry Services (Schjindel, The Netherlands). Diploid *A*. *thaliana* from the “Columbia” line was acquired from the University of Tromsø and provided as a control sample along with the experimental samples. For internal control *Ilex crenata* “Fastigiata” was used.

### 2.3 Analysis of phenotypic responses to different treatments

**FIGURE 2:**
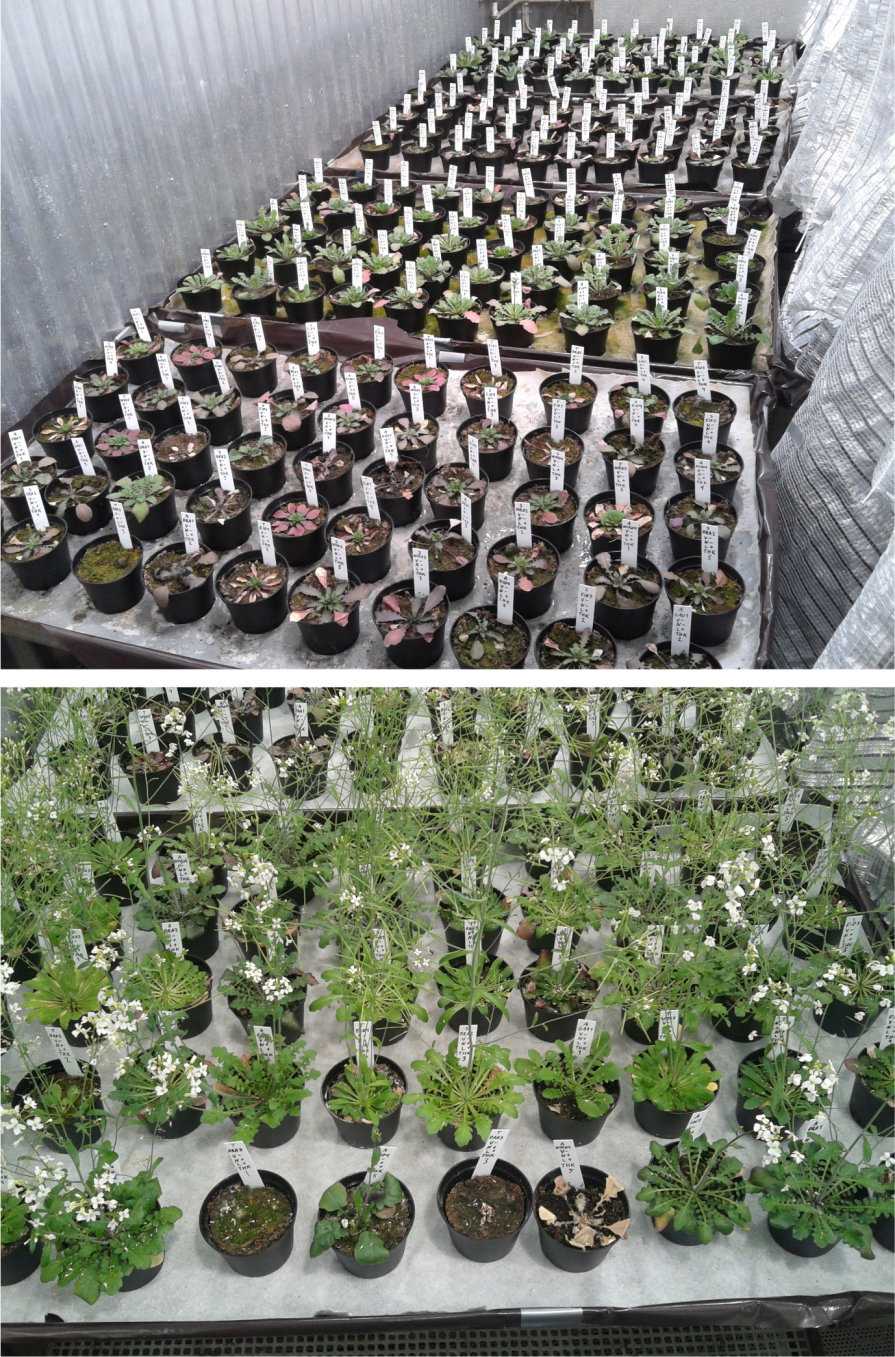
Pictures of plants in the growth chamber experiment. The light‐shading fabric trolleys can be seen to the right in the pictures. Top: Right after vernalization conditions were ended. Bottom: Near the end of the experiment. Photos: T. Kornstad

Seeds from the 10 sampled populations were grown under controlled environmental conditions in a growth chamber. To assess whether different species react differently to varying environmental conditions, eight different treatments were applied in a 2^3^ factorial design. These treatments consisted of all different combinations of dry and wet water conditions, rich and poor nutrient conditions, and high and low light conditions. Five replicates were grown per treatment combination. This adds up to 10 populations × 8 treatment combinations × 5 replicates = 400 plants grown in total. Pictures of the experimental design are shown in Figure 2.

Eight trolleys with a size of 100 × 60 cm were covered first with plastic, and then with felt mats to transport the water evenly over the whole trolley. 50 circular 8C‐101 flowerpots with a diameter of 8 cm (Billund Potter, Billund, Denmark) were placed on each trolley. 400 flowerpots were prepared overall. Each flowerpot was filled with Gartnerjord soil (Tjerbo Torvfabrikk, Rakkestad, Norway) consisting of 86% *Sphagnum* peat, 10% sand and 4% granule clay. One trolley was assigned to each treatment combination. For each population, seeds from all sampled individuals were mixed on a white paper sheet, then several seeds were drawn randomly and sown in each pot. The different populations were distributed randomly within each trolley. 9 l of water were applied to each trolley after sowing.

The seeds were stratified for four days in 4xC and 24 h darkness. Then, conditions were changed to 20°C/17°C day/night temperature and an 8/16 h light/dark cycle. Light was provided by OSRAM 400W Powerstar HQ1^®^‐BT 400W/d Pro Daylight E40 (OSRAM Licht AG, Munich, Germany) light bulbs in GAVITA GAN 400 AL lamps (GAVITA AS, Andebu, Norway). The amount of light in the chamber was measured to 210–250 µmol m^−2^ s^−1^ with a LI‐189 quantum/radiometer/photometer (LI‐COR Biosciences, Lincoln, Nebraska, USA). The seeds were allowed to germinate at similar conditions for all trolleys, and water was applied regularly to avoid desiccation. Almost all seeds from the T‐DRA3 population failed to germinate, and the population was excluded from the experiment. The flowerpots assigned to T‐DRA3 plants were left on the trolleys during the whole experiment, to keep the flowerpot pattern equal between all trolleys. Leaving out the T‐DRA3 population, a total number of 360 plants distributed on the remaining populations were grown for the experiment. Among these, four died during the experiment and were not included in the analyses.

When the seedlings had reached the stadium where primary leaves started to become visible, they were thinned so that one plant remained in each flowerpot. For some populations, transplantations between pots were done. The plants were allowed one week of optimal growth conditions before treatments were applied.

Nutrient treatment was applied by giving nutrient solution made from 1.25 ml Superba NPK 14–4–21 + mikro (Nordic Garden AS, Stokke, Norway) and 1 l water to each of the rich nutrient trolleys once per week, while no nutrients were applied to the poor nutrient trolleys. The water used for making the nutrient solution was included in the total amount of water given to the plants, as described below.

Light treatment was applied by covering the low‐light treatment trolleys with XLS 17 Revolux light‐reducing fabric (AB Ludvig Svensson, Kinna, Sweden). The fabric is partly made from aluminum, and it does not change the spectral composition of the light that passes through. The amount of light below the fabric was measured to be 80–90 µmol m^−2^ s^−1^, equivalent to a reduction of 60–70%.

Water treatment was initially performed by applying 2 l of water three times a week to the wet condition trolleys, and 1 l of water three times a week to the dry condition trolleys. The light‐shading fabric was found to heavily reduce evaporation from the low‐light trolleys, so to obtain similarity in water conditions between the low‐light and the high‐light trolleys, the low‐light trolleys were watered once a week, applying 2 l of water to the wet condition trolleys and 1 l of water to the dry condition trolleys.

Vernalization was initiated 39 days after sowing (35 days after germination conditions were initiated). Growth conditions were changed to 4°C constant temperature and an 8/16 h light/dark cycle. Since growth was low during vernalization, nutrients were applied on average every third week, in the same doses as described above. The amount of light in the growth chamber was reduced to avoid the plants dying from light stress. The amount of light was measured to be 125–135 and 27–32 µmol m^−2^ s^−1^ for the high light and low light treatments, respectively. For the high‐light trolleys, watering was done by applying 2 l of water once a week to the wet condition trolleys, and 1 l of water once a week to the dry condition trolleys. For the low‐light trolleys, watering was done by applying 2 l of water once every third week to the wet condition trolleys, and 1 l of water once every third week to the dry condition trolleys.

Based on findings in Lewandowska‐Sabat et al. (2012), vernalization conditions were kept for 9 weeks. At the end of vernalization, 102 days after sowing, growth conditions were changed to 23°C/20°C day/night temperature and 16/8 h light/dark cycle to allow flowering. Nutrients, light and water treatments were the same as before vernalization. These conditions were kept for 33 days, when the growth experiment was ended.

During the whole experiment, the trolleys were moved around within the chamber, and pots were moved around on the trolleys periodically to avoid edge effects. This was performed haphazardly.

#### 2.3.1 Measurements of phenotypic variables

Phenotypic variables were measured at different times. At the initiation of vernalization, three different variables were measured: number of rosette leaves per plant, length of the longest rosette leaf for each plant (including both petiole and lamina), and length of the lamina on the longest rosette leaf. In cases where leaves were serrated, the length from the innermost serration to the leaf tip was measured and recorded as lamina length.

At the end of the vernalization period, the days it took for each individual plant to bolt and to open the first flower were counted, with the last day of vernalization set as day zero. In addition, the number of rosette leaves was measured at bolting. For plants that bolted, but did not flower, the time to flowering was set as missing data. For plants that neither did flower nor bolt, rosette leaves were counted at the ending day of the experiment, and both time to bolting and time to flowering were set as missing data.

At the end of the experiment, five different variables were measured for each plant: Plant height measured as the longest stem from root to tip for each plant, number of branches on the stem, total number of flowers and siliques (denoted as “number of flowers”—buds were not counted) and dry weight of the aboveground biomass. To measure the dry weight, the plants were harvested and dried at 60°C for 24 h in a TS8136 drying oven (Termaks, Bergen, Norway) before weighing them with AG ED224S scales (Sartorius AG, Groettingen, Germany).

### 2.4 Analysis of genetic markers

For genetic analyses, seeds from each plant harvested during the fieldwork were sown in individual pots for the populations A‐GAU1, A‐NFRO4, A‐DRA1, S‐DRA2, S‐NFRO3, S‐DRA2, S‐EID3, T‐SFRO3, and T‐EID1. For T‐DRA3, seeds harvested from the plants grown in the growth chamber experiment were sown. A‐EID4 was not available for analysis, since there were very few viable seeds left. The plants were grown until large enough to permit harvesting. Fresh tissue was harvested from one individual per pot. No standardization was done when it came to harvesting. During the harvest, ~100 µg of fresh tissue per plant was cut in pieces with scissors and put into 2‐ml tubes (Eppendorf). The tubes were stored at −80°C. Before isolation of DNA, two 3 mm crushing beads were applied to each tube. The tubes were dipped into liquid nitrogen, before the tissue was crushed with a TissueLyser II (QIAGEN, Hilden, Germany) for 1 min at 20 r/s. DNA was extracted from the crushed tissue using a DNeasy Plant Mini Kit (QIAGEN). The quantity of DNA in each isolation was checked using a NanoDrop 8000 UV‐Vis Spectrophotometer (Thermo Fisher Scientific, Waltham, Massachusetts, USA). A modified AFLP protocol after Hayashi et al. (2005) and Vos et al. (1995) was run on the genomic DNA. Briefly, 400 ng genomic DNA, 1 x RL buffer (100 mM trisHAc, 100 mM MgAc, 500 mM Kac, 50 mM DDT), 0.05 µg Bovine Serum Albumin (BSA, New England BioLabs, Ipswich, MA, USA), 0.125 units *EcoR*I enzyme (Invitrogen, Carlsbad, CA, USA), and 0.125 units *Mse*I enzyme (New England Biolabs) was adjusted with MilliQ water to a total of 40 µl and incubated at 37°C for 75 min. Adapters (Invitrogen) were annealed by mixing forward (*EcoR*I‐F 5'‐ CTC GTA GAC TGC GTA CC ‐3', *Mse*I‐F 5’‐ GAC GAT GAG TCC TGA G ‐3') and reverse adapters (*EcoR*I‐R 5'‐ AAT TGG TAC GCA GTC TAC ‐3', *Mse*I‐R 5'‐ TAC TCA GGA CTC AT ‐3') to a concentration of 10 µM (*EcoR*I adapters) or 50 µM (*Mse*I‐adapters) followed by incubation at 65°C for 10 min, 37°C for 10 min and 25°C for 10 min. 0.1 µM annealed *EcoR*I adapter, 1 µM *Mse*I adapter, 0.2 µM ATP (Sigma Aldrich, St. Louis, MO, USA). 1xRL‐buffer, 0.05 µg BSA and 0.02 units T4 DNA ligase (Invitrogen) was added to the restricted DNA and adjusted with MilliQ water to a total of 50 µL and incubated at 37°C for three hours. The ligated DNA was diluted 10x with MilliQ water.

One E+1 primer (E01) and one M+1 primer (M01) was used for preamplification. MilliQ water, 1× PCR buffer (Applied Biosystems), 2 mM MgCl2 (QIAGEN, Hilden, Germany), 0.2 mM dNTP (Invitrogen), 0.3 μM E+1 primer (Invitrogen), 0.3 μM M+1 primer (Invitrogen) and 0.038 units of AmpliTaq DNA polymerase (applied and 3 µl diluted restricted and ligated DNA was mixed to a volume of 13 μe. PCR was run with the following program: 94°C for 2 min, then 20 cycles of 94°C for 20 s, 56°C for 30 s, and 72°C for 2 min, then 72°C for 2 min, then 60°C for 30 min. The preamplified DNA was diluted 10× with MilliQ water.

MilliQ water, 1 μl 1× PCR buffer (QIAGEN), 0.5 mM MgCl2 (QIAGEN), 0.2 mM dNTP (Invitrogen), 0.625 μM fluorescently labeled E+3 primer (Invitrogen), 0.625 μM M+3 primer (Invitrogen), and 0.025 units of HotStarTaq DNA polymerase (QIAGEN) and 2.5 μl of diluted preamplified DNA was adjusted to a total volume of 10 μl. PCR was run using the following program: 95°C for 15 min, then 10 cycles of 94°C for 20 s, 66°C for 30 s with a reduction of 1°C per cycle and 72°C for 2 min, then 25 cycles of 94°C for 30 s, 56°C for 30 s and 72°C for 3 min, then 60°C for 30 min. Six different combinations of E+3(fluorescently labeled) and M+3 primers were tested for selective amplification (E33xM36, E33xM37, E33xM38, E42xM36, E42xM37, E42xM38). The three underlined combinations yielded the best testing results based on the number of amplified fragments in the range 50–500 base pairs, and amount of polymorphism among the included individuals and were chosen for further analyses.

#### 2.4.1 μL Hi‐Di™ formamide (Life Technologies, Carlsbad, CA, USA), 0.05 μl GeneScan™

500 LIZ^®^ Size Standard (Life Technologies) and 1 μL diluted amplified DNA was mixed and denatured for 3 minutes at 95°C. Electrophoresis was performed with an ABI PRISM 3730 DNA analyzer (Applied Biosystems).

The AFLP results were scored using GeneMapper^®^ ver. 5.0 (Life Technologies). Only single peaks clearly differentiated from background noise were scored as a band. Manual corrections were run on all samples. Individuals showing anomalous peak patterns on at least one of the three primer combinations were removed completely from the dataset. The number of replicated samples was 31 (22.7% of the total number of samples) for primer combination E33xM37, 30 (22.1% of the total number of samples) for primer combination E33xM38 and 23 (16.9% of the total number of samples) for primer combination E42xM38. The genotyping error, due to for instance incomplete digestion or imperfect PCR, for each primer combination was calculated using the formula (total number of scoring errors) × 100/(number of replicates) × (number of markers) (Bonin et al., 2004), then a final genotyping error was calculated by computing a weighted mean between the primer combinations. Alleles showing a high level of inconsistency between the replicated samples were removed before calculating the genotyping error, and not included in the analyses. The numbers of assessed individuals per population were 6 individuals from A‐DRA1, 18 individuals from A‐GAU1, 17 individuals from A‐NFRO4, 16 individuals from S‐DRA2, 16 individuals from S‐EID3, 20 individuals from S‐NFRO3, 6 individuals from T‐DRA3, 18 individuals from T‐EID1 and 19 individuals from T‐SFRO3.

### 2.5 Data analysis

All data analyses were done with RStudio version 2021.09.2+382 (RStudio, 2021), based on R version 4.1.2 (R Core Team, 2021), unless anything else is specified in the text.

#### 2.5.1 Phenotypic variables

Descriptive multivariate analysis using non‐metric multidimensional scaling (NMDS) from the R package vegan (Oksanen et al., 2020) was run on all measured response variables, to obtain a crude picture of how the different species reacted to the different combinations of treatment. A non‐metric approach was chosen since several of the response variables were non‐linear and/or non‐normal. Some of the variables were discarded from further analysis for different reasons: Days to bolting (correlated with days to flowering, *r* = 0.70), number of leaves at start of vernalization (closely correlated with number of leaves at bolting, *r* = 0.90), number of branches (zero inflated and thus hard to analyze properly), and length of lamina on longest leaf and its percentage of total leaf length (irrelevant variables in an ecological perspective). The remaining variables were superimposed onto a biplot of the first two NMDS axes.

To assess the effect of treatment and species on the different variables, linear mixed effects models or generalized linear mixed effects models were run, using the R packages nlme (Pinheiro et al., 2017) and lme4 (Bates et al., 2007). Water was initially regarded as giving no effect, but an assessment of model selection criterions (data not shown) found that including water gave slightly better models. For the final models, a single factor was constructed, where each level corresponded to a specific combination of light, nutrients, water, and species for a total of 2^3^ × 3 = 24 levels. Population was added as a random effect.

To run the models, the response variables biomass and number of flowers were log‐transformed. Days to flowering and number of leaves were considered count data, and Poisson models were used for assessing them. Number of flowers could also be considered count data, but as the numbers were so large, we concluded that the variable should be considered as continuous instead of discrete. Table 2 gives an overview of transformation of variables, and which models that were run for each response variable. Both linear mixed effects models and generalized linear models with Poisson family were fit using maximum likelihood. All models were checked for assumptions of normality and equality of variance between groups by conferring Q‐Q and residual plots. Poisson models were checked for over‐ and underdispersion.

**TABLE 2:** Overview of type of models run for the phenotypic response variables, including eventual transformation or GLMM family. LMM = Linear mixed model, GLMM = Generalized Linear Mixed Model

Response	Type of model	Transformation	GLMM family
Biomass	LMM	Natural logarithm	—
Days to flowering from vernalization	GLMM	—	Poisson
Height	LMM	—	—
Number of leaves at bolting	GLMM	—	Poisson
Number of flowers	GLMM	Natural logarithm	—
Longest leaf at start of vernalization	LMM	—	—

Post hoc testing of the models was done by applying general linear hypothesis methods from the R package multcomp (Hothorn et al., 2008). These methods give a generalization of the Tukey post hoc test that can be used on unbalanced designs. To model reaction norms for each species to the applied treatments, common letter displays based on multiple comparisons between all pairs were constructed.

To check whether the species reacted differently to environmental stress and showed differences in phenotypic plasticity, confidence intervals for estimated differences between high and low levels of treatments were constructed. Nutrient effects were assessed separately for each combination within the two other treatments. A corresponding approach was used for light and water effects. To adjust for multiple comparisons, a confidence level of 99% were used in the post hoc analyses.

Coefficients of variation were calculated for each response variable. The measurement gives an indication on the amount of phenotypic plasticity (Schlichting & Levin, 1984; Sultan, 2001). Variables were not transformed for this calculation. The formula used for calculation was $100*sd\left({\bar{X}}_{i}\right)/mean\left({\bar{X}}_{i}\right)$, where *i* denotes the different treatment levels. This was done both on the population and on the species level. Estimates of uncertainty were unavailable, meaning that it was not possible to evaluate whether significant differences could be found.

To assess fitness homeostasis in the different species, a comparison variable called *C* comparing experimental variables connected to fitness by Davidson et al. (2011) with other experimental variables connected to phenotypic plasticity was constructed. Variables connected to fitness included number of flowers and total biomass, while variables not connected to fitness included height of plants, number of leaves at the end of the experiment and the length of the longest leaf at the start of vernalization. Some of the variables were transformed to make them more linear: Biomass (natural logarithm), number of flowers (natural logarithm of (number of flowers + 1)) and number of leaves at the end of the experiment (natural logarithm). To make the variables comparable, they were standardized to occupy an interval between 0 and 1. This was achieved by 1) adding/subtracting the lowest number in the variable to all observations in the variable so that the lowest number in the variable would be 0 and 2) dividing all observations by the highest number in the variable. From the transformed and scaled variables, the formula $C=\frac{\text{Flowers}+\text{Biomass}}{\text{Nleaves}+\text{Leaf length}+\text{Height}}$ was used to construct the comparison variable.

A linear mixed model, and general linear hypothesis post hoc methods as described above, were applied to the comparison model. A confidence level of 99% was used for the post hoc analyses. The theory was that a higher value of *C* means relatively more allocation of resources to fitness, and vice versa. A smaller difference in *C* between good and poor environmental conditions could be interpreted as a higher degree of fitness homeostasis.

#### 2.5.2 Population structure and genetic diversity

The dataset was examined for population structure using the software Structure, a software that can allocate individuals to genetic groups based on AFLP data (Pritchard et al., 2000). Analyses were run using Structure ver. 2.3.4 at the Lifeportal, University of Oslo (https://www.uio.no/english/services/it/research/hpc/portals/lifeportalPaleontología Electrónica), with 10^6^ iterations and a burn‐in of 10^5^ iterations. An admixture model was used; meaning that for each individual different parts of the genome are allowed to descend from different groups. Linkage between markers was not considered. A minimum of one population (*K* = 1) and a maximum of 9 populations (*K* = 9) was allowed per analysis. For each value of *K*, 10 independent runs were done. The results were assessed using the R functions in Structure‐sum (Ehrich, 2011). The number of clusters was chosen after an evaluation based on the following criteria: (1) all runs gave similar results, (2) similarity coefficient close to 1.0, (3) highest possible ln P (data) and (4) highest possible ΔK (Evanno et al., 2005; Pritchard et al., 2000). Structure analysis was run for each species. In addition, an analysis incorporating all individuals was run in order to see whether the different species clustered separately.

To visualize the clusters in a multidimensional space, principal coordinate (PCO) analysis was run on a distance matrix calculated with Dice's coefficient of similarity (Dice, 1945). The PCO analyses were run in PAST ver. 2.17c (Hammer et al., 2001), and scores for the two first components were extracted and plotted in R. PCO analysis was run for all species together, and separately for each species.

To assess and compare the diversity of the sampled populations and species, 95% confidence intervals for Nei's Genetic Diversity (Nei, 1987) was constructed using bootstrapping over 1000 replicates with the R functions in AFLPdat (Ehrich, 2006). Analyses of molecular variance (AMOVA) (Excoffier et al., 1992) were performed in Arlequin ver. 3.5 (Excoffier & Lischer, 2010). This was done for each species based on groups inferred from the original populations. If the number of clusters inferred from Structure came out differently from the original populations, an additional AMOVA was run based on the inferred clusters (unless the inferred number of clusters was one).

#### 2.5.3 Comparison of genetic diversity and phenotypic plasticity

To assess possible relationships between genetic diversity and phenotypic plasticity, a Mantel test was run to compare Euclidean distance matrices calculated from Nei's Genetic Diversity and Coefficients of variation for all phenotypic variables. The test was run on the eight populations where results from both growth experiments and genetic analyses were available. A corresponding test was also done with a phenotypic distance matrix calculated from coefficients of variation where each variable was scaled to unity. The scaling was done by dividing all values in the variables by the highest value in the variable.

## 3 RESULTS

### 3.1 Measurements of ploidy level and chromosomal numbers

The populations used in the experiment mainly showed the expected chromosomal numbers and ploidy levels: 10 chromosomes/diploid for *A*. *thaliana*, 32 chromosomes/tetraploid for *A*. *arenosa* and 26 chromosomes/tetraploid for *A*. *suecica*. There were two exceptions: One individual showed a lower chromosomal number than expected in *A*. *arenosa*. This might be due to aneuploidy, but it might also be due to errors in the measurement. One alleged individual *A*. *thaliana* showed a chromosome number that one would expect for *A*. *arenosa*. This is probably due to a confusion of samples. The samples used for flow cytometry were not used in the other experiments, hence this likely did not affect the rest of the results.

### 3.2 Growth experiment

#### 3.2.1 Multivariate analysis of phenotypic variables

**FIGURE 3:**
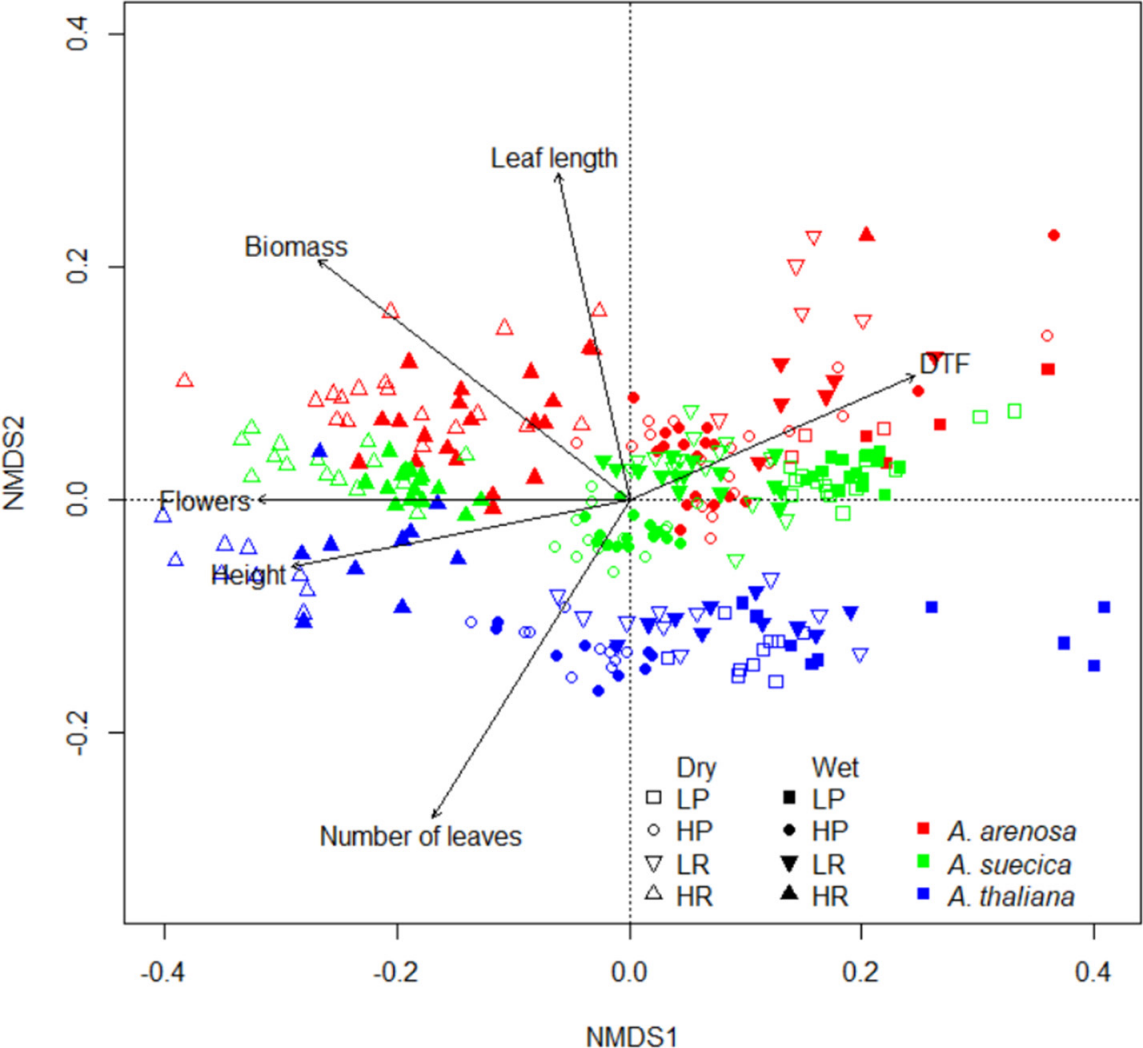
Biplot of the two first NMDS axes, showing all observations grouped after species and treatment. LP = low light, poor nutrients, HP = high light, poor nutrients, LR = low light, rich nutrients, HR = high light, rich nutrients. The arrows show the phenotypic response variables and what trends they exhibited

Clustering of phenotypic variables in response to light and nutrients show that *A*. *thaliana* was separated from the other two species (Figure 3). There was a weak trend that *A*. *suecica* occupied the space between *A*. *arenosa* and *A*. *thaliana*. There was not a clear clustering between treatments, although rich nutrients and high light tended to cluster on the top left side of the plot. This indicates that rich nutrients and high light were associated with taller plants, higher biomass and more flowers. For water, no clustering tendencies were observed. For all variables included in the NMDS, *R* ^2^ were >0.50 and *p*‐values were <0.001.

#### 3.2.2 Analyses of phenotypical variables

**FIGURE 4:**
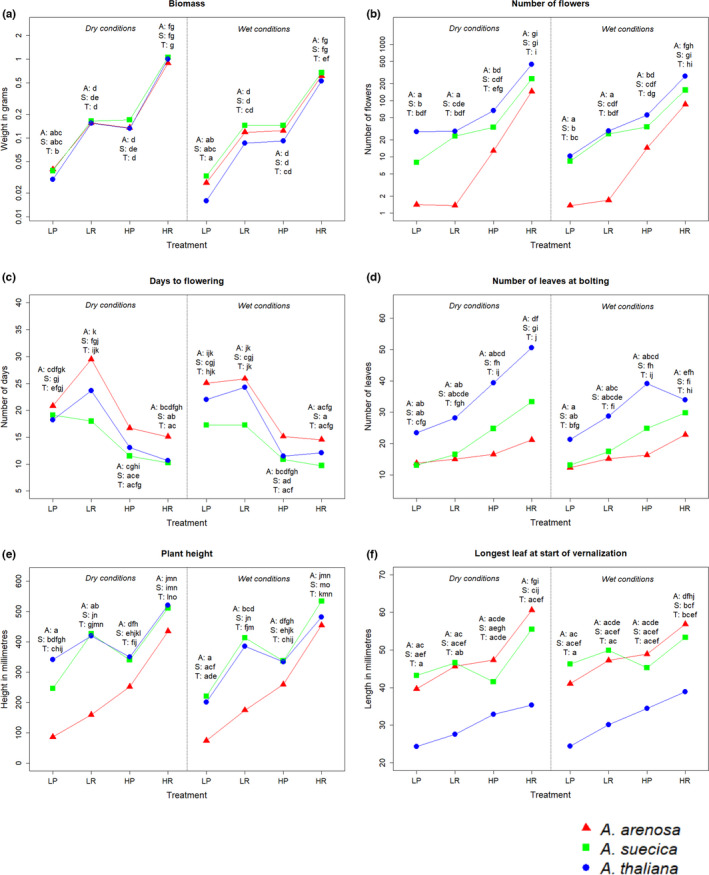
Assessments of reaction norms within the different species. The data represents means within the given species and treatment combination. For biomass, flowers, days to flowering, and numbers of leaves at bolting, means were calculated from log‐transformed data. Common letters denote no significant difference, on a 99% confidence level. A (red) = *A*. *arenosa*, S (green) = *A*. *suecica*, T (blue) = *A*. *thaliana*. LP = low light, poor nutrients, LP = low light, nutrient‐poor, LR = low light, nutrient‐rich, HP = high light, nutrient‐poor, HR = high light, nutrient‐rich. (a) Biomass (dry weight in g), (b) Number of flowers, (c) Days to flowering after vernalization, (d) Number of leaves at bolting, (e) Plant height at harvest (in mm), (f) Longest leaf at start of vernalization (in mm)

The general trend was an increase in the measurements of phenotypic variables from low light, poor nutrients via low light, rich nutrients/high light, poor nutrients to high light, rich nutrients (Figure 4). The exception from this was days to flowering (DTF), where the trend was the opposite. This is expected, since plants are anticipated to flower faster when conditions are better.

Reaction norms differed between species in some of the response variables (Figure 4). Biomass response to changing light and nutrient conditions were similar between all three species, under both dry and wet conditions. However, *A*. *thaliana* showed a weak trend toward producing less biomass than the other two species under wet conditions (Figure 4a). There was a tendency that *A*. *arenosa* plants were shorter and produced fewer flowers than the other two species. This was significant for the low light treatments (Figure 4b,e). Under most of the treatments *A*. *suecica* showed a tendency to flower later than the two other species, but this was not significant (Figure 4c). *Arabidopsis thaliana* plants had more leaves than the other species at the time of bolting under dry conditions, while the tendency was less clear under wet conditions (Figure 4d). *Arabidopsis suecica* was intermediate to the parent species when it came to number of flowers, number of leaves at bolting and partially in longest leaf at start of vernalization (Figure 4b,d,e).

**FIGURE 5:**
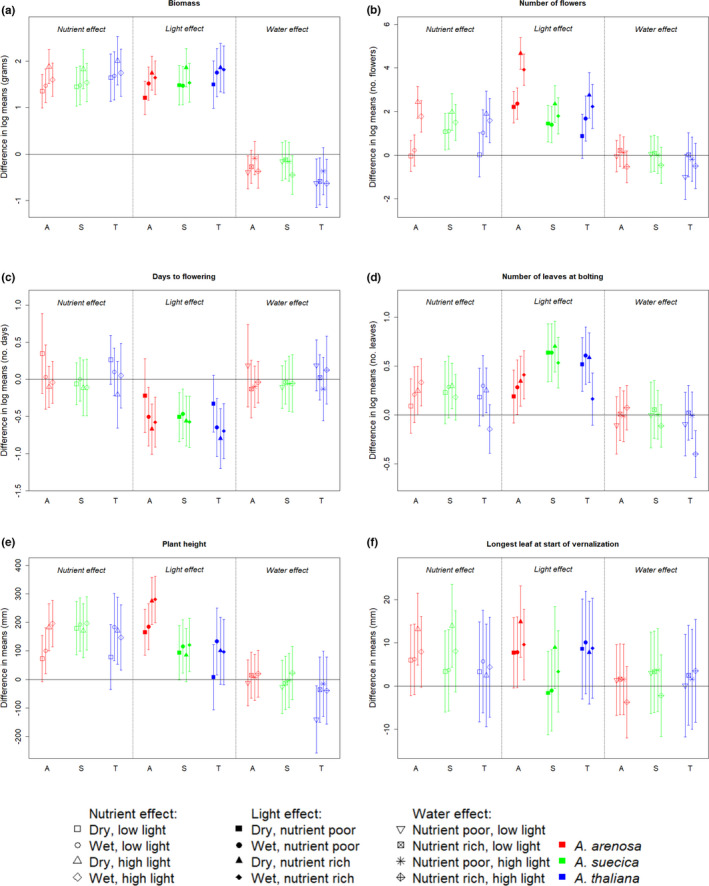
99% confidence intervals for estimates of phenotypic plasticity in the different species, as a response to the different treatments. The symbols show estimated differences between high and low levels, while the error bars show the confidence intervals. For each treatment, effects are considered within the different combinations of the other treatments, as shown in the legend. For biomass, flowers, days to flowering, and numbers of leaves at bolting, differences in log‐transformed data are shown. A (red) = *A*. *arenosa*, S (green) = *A*. *suecica*, T (blue) = *A*. *thaliana*. (a) Biomass (dry weight in g), (b) Number of flowers, (c) Days to flowering after vernalization, (d) Number of leaves at bolting, (e) Plant height at harvest (in mm), (f) Longest leaf at start of vernalization (in mm)

Both nutrient and light treatments induced significant positive differences in biomass between high and low levels. Meanwhile, water treatment showed a tendency towards negative differences in biomass between high and low levels, but this was not significant for all species and treatment combinations (Figure 5a). *Arabidopsis thaliana* showed the strongest trend toward negative response to wet conditions.

For the other measured variables, the effects were more unclear. For number of flowers, the light treatment mainly showed significant positive differences. In *A*. *arenosa* an interaction effect between nutrients and light was observed: The positive differences in number of flowers between high and low levels of light were significantly higher under nutrient‐rich conditions, and correspondingly, the differences between nutrient‐rich and nutrient‐poor conditions were higher under high light conditions (Figure 5b).

The light treatment also mainly showed effect on days to flowering, and number of leaves at bolting, but this was not significant for all species and treatment combinations (Figure 5c,d). Both nutrient and light seemed to influence plant height, for light the tendency seemed stronger in *A*. *arenosa* (Figure 5e). For the longest leaf at the start of the vernalization, few and weak significant effects were found (Figure 5f).

Overall, very few significant differences were observed between dry and wet conditions (Figure 3), meaning that the water treatment provoked few effects in the experiment.

*Arabidopsis arenosa* seemed to exhibit larger phenotypic plasticity when it comes to height and number of flowers, while *A*. *suecica* differed from the other species when it comes to leaves at bolting (Figure 4; Table 3). The variation was large on the population level, but the general trends from the species level were reflected in the populations.

**TABLE 3:** Estimated coefficients of variation for the phenotypic response variables, measured across species and populations

Species	Biomass	Flowers	DTF	Leaves at bolting	Height	Longest leaf
*A. arenosa*	116.71	162.12	28.65	21.47	61.03	14.83
*A. suecica*	120.68	129.89	27.99	35.46	29.94	10.06
*A. thaliana*	130.12	125.85	34.13	29.08	25.98	17.01
Population						
A‐DRA1	118.16	167.52	28.21	27.37	50.84	13.08
A‐EID4	137.04	165.67	33.91	25.07	71.72	24.29
A‐GAU1	109.13	151.67	30.32	18.41	67.64	16.17
A‐NFRO4	116.65	170.17	35.29	20.23	68.06	19.80
S‐DRA2	124.07	126.36	22.95	31.04	27.35	10.77
S‐EID3	122.76	136.07	28.77	38.03	31.18	13.04
S‐NFRO3	114.91	130.94	34.21	38.44	33.70	12.89
T‐EID1	133.58	139.63	39.67	29.99	38.46	17.52
T‐SFRO3	133.76	120.12	30.21	33.33	16.51	21.57

**FIGURE 6:**
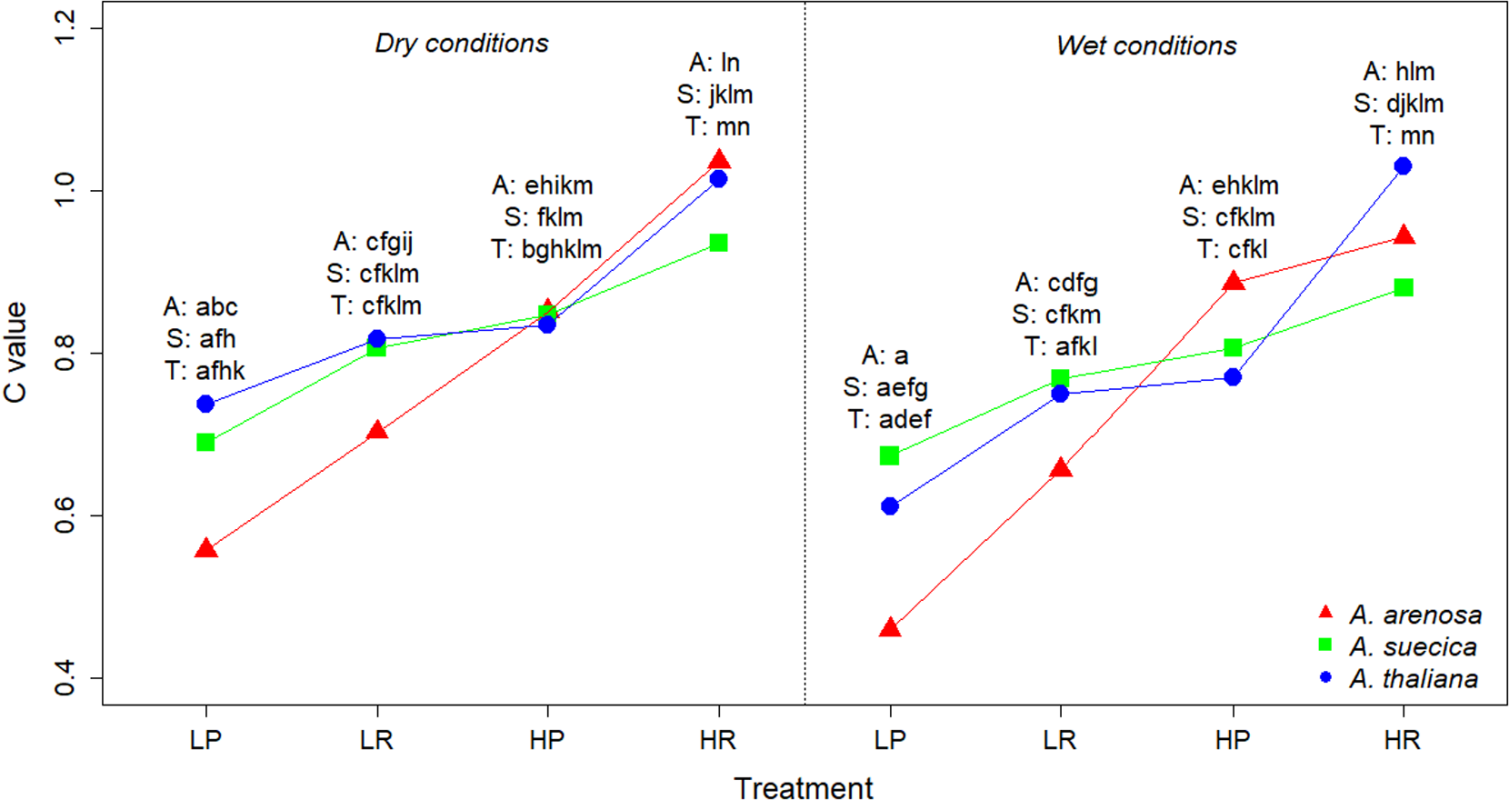
Reaction norm for the comparison variable *C* (which compares response variables connected to fitness with other response variables connected to phenotypic plasticity) within the different species. Common letters denote no significant difference, on a 99% confidence level. A (red) = *A*. *arenosa*, S (green) = *A*. *suecica*, T (blue) = *A*. *thaliana*. LP = low light, nutrient‐poor, LR = low light, nutrient‐rich, HP = high light, nutrient‐poor, HR = high light, nutrient‐rich

In the analysis of the comparison variable *C* (which compares response variables connected to fitness with other response variables connected to phenotypic plasticity), the species had similar values within the high light treatment (Figure 6). Within the low light treatment, *A*. *arenosa* seemed to exhibit lower values than the other two species, although this trend was not significant. Still, this could indicate that *A*. *arenosa* allocates fewer resources to keep up fitness under low light treatments than the other two species. Responses in the *C* variable between high and low treatment levels were not significantly different between species within any of the treatments (data not shown).

### 3.3 Analyses of population structure and genetic diversity

A total number of 136 individuals were analyzed for variation in 274 AFLP markers (100 E33xM37 markers, 97 E33xM38 markers and 77 E42xM38 markers). 63 markers were only present in *A*. *arenosa*, 27 were only present in *A*. *suecica*, 16 were only present in *A*. *thaliana*, 67 were present in *A*. *arenosa* and *A*. *suecica*, 45 were present in *A*. *suecica* and *A*. *thaliana*, 12 were present in *A*. *arenosa* and *A*. *thaliana* and 44 were present in all three species. The percentage of polymorphic markers was 95.2% in *A*. *arenosa*, 82.5% in *A*. *suecica* and 77.8% in *A*. *thaliana*. The genotyping error was calculated to be 3.30%.

#### 3.3.1 Population structure

**FIGURE 7:**
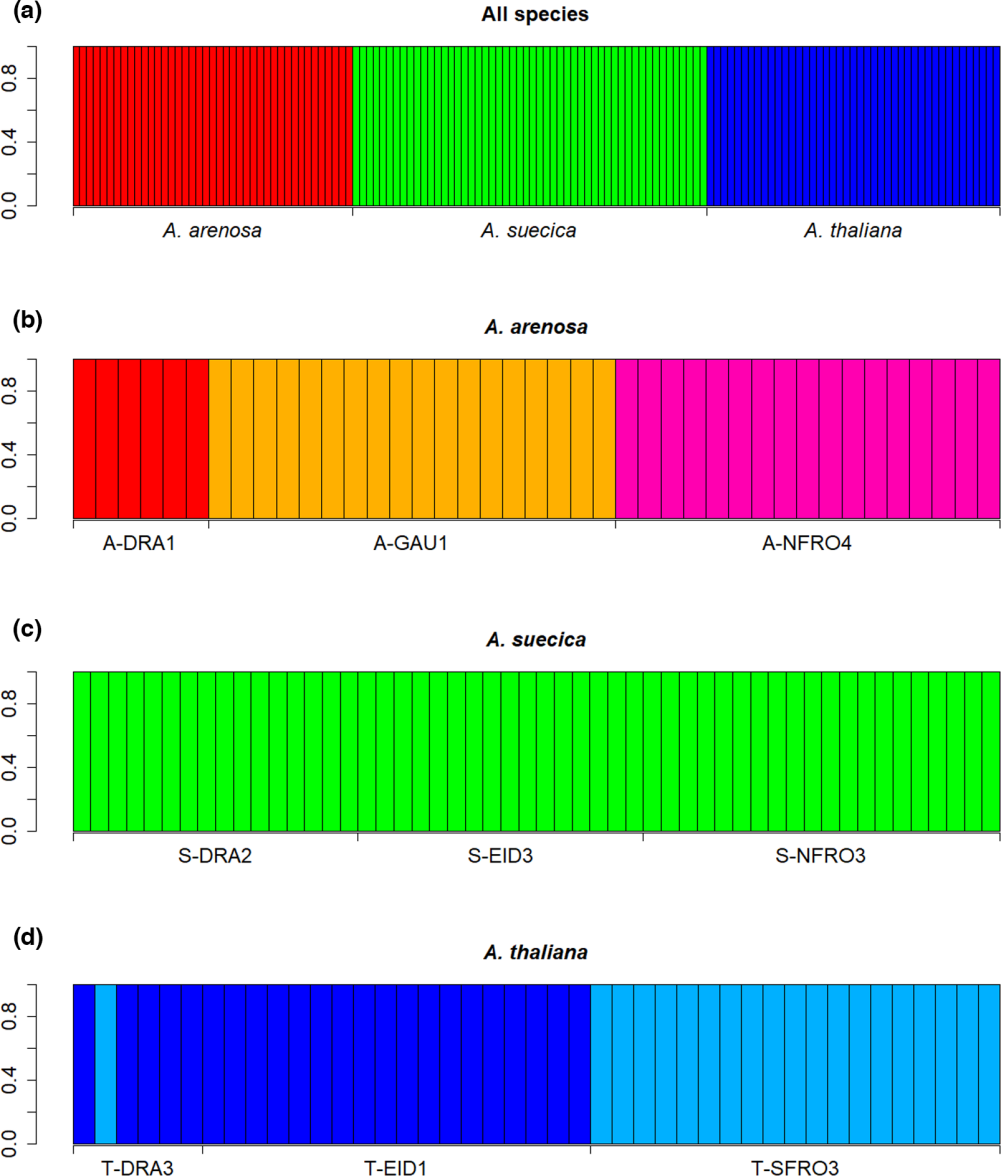
Bar plots showing allocations to clusters from Structure. The vertical axis denotes probability of allocation to a cluster. (a) Analysis of all individuals (*K* = 3), (b) Analysis of *A*. *arenosa* (*K* = 3), (c) Analysis of *A*. *suecica* (*K* = 1), (d) Analysis of *A*. *thaliana* (*K* = 2)

**FIGURE 8:**
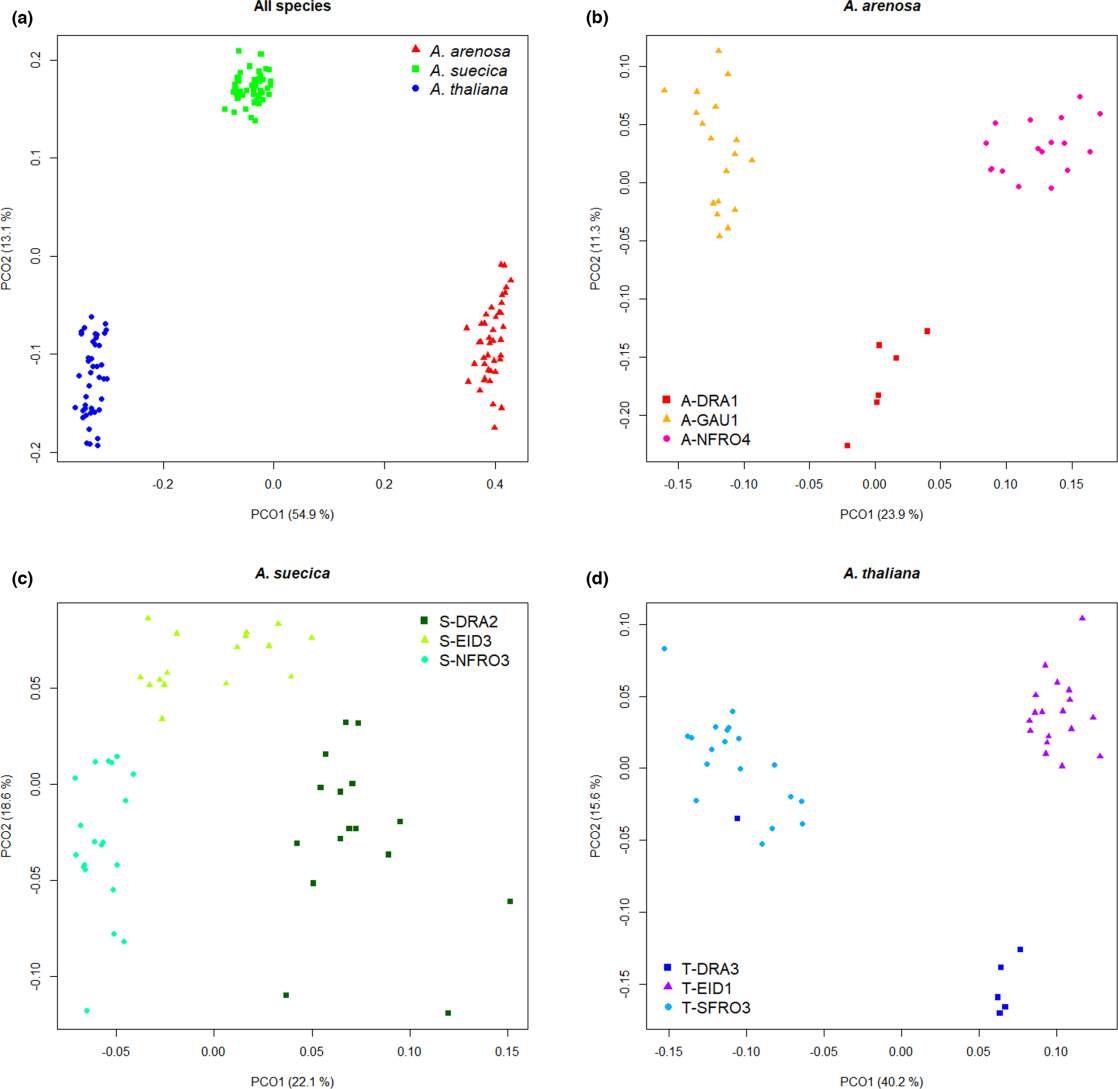
Plots showing scores on the first and second PCO components from PCO analyses on Dice distances between AFLP markers. (a) All species (red = A. *arenosa*, green = A. *suecica*, blue = A. *thaliana*), (b) *A*. *arenosa* (red = A‐DRA1, orange = A‐GAU1, pink = A‐NFRO4), (c) *A*. *suecica* (dark green = S‐DRA2, light green = S‐EID3, turquoise = S‐NFRO3), (d) *A*. *thaliana* (blue = T‐DRA3, purple = T‐EID1, light blue = T‐SFRO3)

**FIGURE 9:**
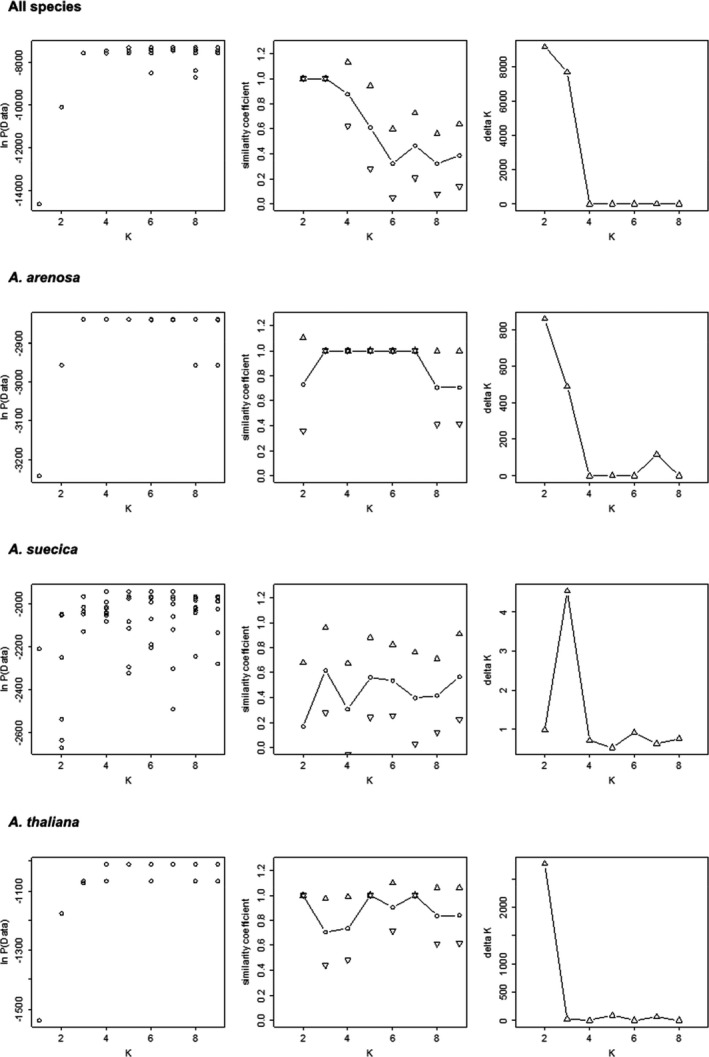
Basis for selection of optimal number of clusters (K) in Structure

The results from Structure showed a clear clustering of the different species (Figure 7a). This was confirmed by the PCO (Figure 8a), where we also see that *A*. *suecica* was placed in the middle of the first axis between its parent species. On the population level, *A*. *arenosa* showed a clear population clustering both in Structure and PCO (Figures 7b and 8b). In *A*. *suecica* no clear population structure was found (Figure 7c), but the PCO indicated a clustering of the different populations (Figure 8c). In *A*. *thaliana*, Structure identified one cluster consisting of T‐SFRO3 and one cluster consisting of T‐DRA3 and T‐EID1 (Figure 7d). One individual in T‐DRA3 clustered with T‐SFRO3, and this was reflected in the PCO plot (Figure 8d). This individual was removed before analyzing genetic diversity and running AMOVA. In a structure analysis run only on T‐DRA3 and T‐EID1 without the misplaced individual, all individuals clustered to their respective populations (data not shown). No individuals showed mixed descent within any of the Structure analyses. Figure 9 shows the graphs that underlie the decisions on optimal numbers of clusters.

#### 3.3.2 Genetic diversity

**FIGURE 10:**
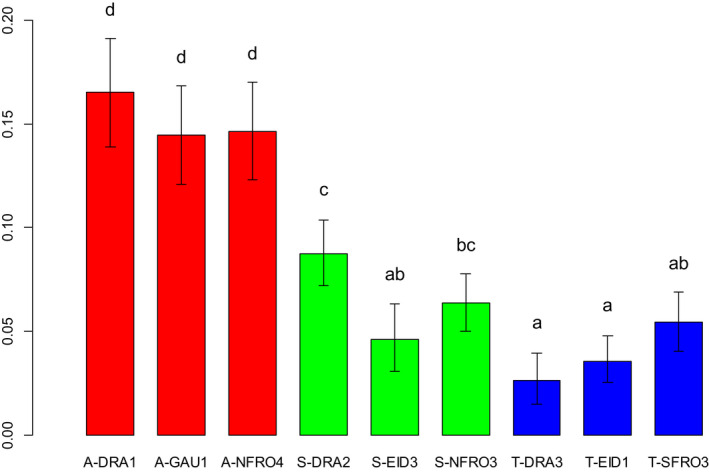
Barplot of Nei's Genetic Diversity within the investigated populations. The vertical axis shows the diversity measure. Error bars denote 95% confidence intervals, calculated using bootstrapping over 1000 replicates and all AFLP markers. Common letters denote populations that are not significantly different from each other

**FIGURE 11:**
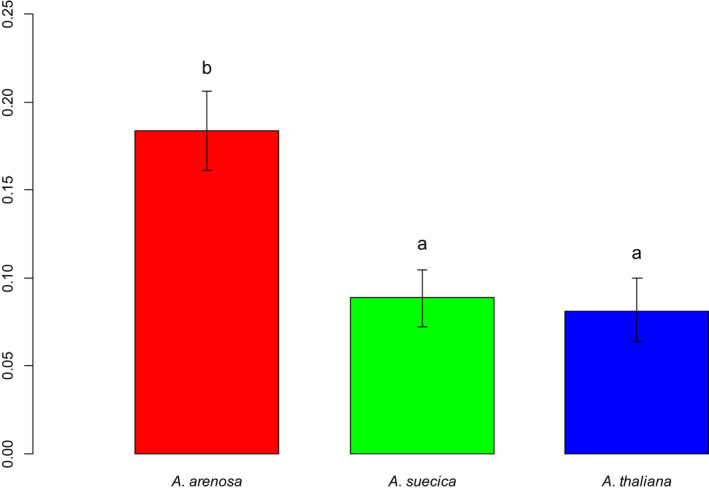
Barplot of Nei's Genetic Diversity within the investigated species. The vertical axis shows the diversity measure. Error bars denote 95% confidence intervals, calculated using bootstrapping over 1000 replicates and all AFLP markers. Common letters denote species that are not significantly different from each other

The *A*. *arenosa* populations exhibited significantly higher genetic diversity than the *A*. *suecica* and *A*. *thaliana* populations (Figure 10), and this was confirmed on the species level (Figure 11). Two of the *A*. *thaliana* populations (T‐DRA3 and T‐EID1) exhibited the lowest genetic diversity. For T‐DRA3, the number of sampled individuals was so low that the total sample did not necessarily reflect the population diversity. More diversity was observed within *A*. *suecica* populations than within *A*. *thaliana* populations (Figure 10). No significant difference could be found between *A*. *suecica* and *A*. *thaliana* on the species level (Figure 11).

#### 3.3.3 Analysis of molecular variance (AMOVA)

The AMOVA showed that the between‐populations percentage of variation in the AFLP markers was 27.5% in *A*. *arenosa*, 34.5% in *A*. *suecica* and 58.8% in *A*. *thaliana* when considering the original populations (Table 4). When considering *K* = 2 clusters in *A*. *thaliana*, the between‐population percentage of variation was still quite high (48.6%).

**TABLE 4:** Analysis of molecular variance (AMOVA) on (a) Populations within *A*. *arenosa*, (b) Populations within *A*. *suecica*, (c) populations within *A*. *thaliana* and (d) clusters within *A*. *thaliana* inferred from the *K* = 2 Structure analysis. *p*‐values for all estimations are <0.001

Species	Source of variation	d.f.	Sum of squares	Variance components	Percentage of variation
(a) *A*. *arenosa*	Among populations	2	234.480	7.704	27.52
	Within populations	38	770.935	20.288	72.48
(b) *A*. *suecica*	Among populations	2	180.178	4.709	34.47
	Within populations	49	438.688	8.953	65.53
(c) *A*. *thaliana* on populations	Among populations	2	224.049	8.456	58.82
	Within populations	39	230.856	5.919	41.18
(d) *A*. *thaliana* on *K* = 2 clusters	Among clusters	1	155.028	7.090	48.60
	Within clusters	40	299.876	7.497	51.40

### 3.4 Comparison of genetic diversity and phenotypic plasticity

The Mantel test showed a significant positive correlation between phenotypic plasticity measured as coefficients of variation and the measurements of Nei's Genetic Diversity (Table 5). This indicates that there is a relationship between higher genetic diversity and larger phenotypic plasticity on the population level among the study species. The corresponding test done with a distance matrix created from coefficients of variation scaled to unity also yielded a significant positive correlation (Table 5).

**TABLE 5:** Results from Mantel tests comparing distance matrices constructed from unscaled and scaled coefficients of variation from Table 3 with the distance matrix constructed from the estimates of Nei's Genetic Diversity presented in Figure 11. The table shows estimates, lower and upper bound for 95% confidence intervals and p‐values for correlation

	Estimate	Lower bound 95% CI	Upper bound 95% CI	*p*‐Value
Unscaled	0.697	0.627	0.806	0.020
Scaled	0.545	0.408	0.746	0.018

## 4 DISCUSSION

### 4.1 *Arabidopsis suecica* is intermediate to its parent species in both pheno‐ and genotype

The species are clearly separated based on genetic analyses and there is no sign of hybridization between them (Figures 7a and 8a). *Arabidopsis suecica* is intermediate between *A*. *thaliana* and *A*. *arenosa* (Figure 8a), reflecting its status as an allopolyploid offspring species. This is also seen in the phenotypic analysis (Figure 4), although the tendency is not as clear as it is for the genotypic.

No clear population structure could be found in *A*. *suecica*, even though inbreeding species are expected to exhibit more genetic structure among populations than outcrossing species (Loveless & Hamrick, 1984). All investigated populations of *A*. *suecica* were found along railway lines, indicating that most of the Norwegian *A*. *suecica* consists of a large, coherent population. *Arabidopsis thaliana* is at least partly indigenous in Norway, whereas *A*. *arenosa* and *A*. *suecica* are introduced. It is plausible that the railway populations of *A*. *suecica* in Norway have a single, recent common ancestor, and that there has not been enough time for a clear population structure to develop. The AMOVA results show that the between‐population variation was much higher in *A*. *thaliana* than in the two other species (Table 4), probably due to *A*. *thaliana* having developed genetic differentiation over a longer time period than the other species.

Most of the phenotypic variables show common trends among the species (Figures 4 and 5). However, *A*. *arenosa* differed from the other species in several response variables when it came to response to light treatment. The reason for this might be found in the species’ life histories. *Arabidopsis arenosa* requires insect pollination, while the two other species are selfers (Säll et al., 2004). The amount of light could thus have less impact on the selfing species’ ability to reproduce successfully. Nutrient availability gives similar responses in all three species. In the wild, the species tend to grow in sandy, nutrient‐poor soil (Elven, 2005). The similar response patterns suggest that they thrive under poor conditions but have the capability to behave opportunistically when nutrient availability improves. Few responses were observed between differing watering regimes, possibly because the applied treatments did not concur with what could be classified as high and low levels of water for *Arabidopsis* species.

### 4.2 *Arabidopsis arenosa* responds most strongly to changing environments and shows the highest level of genetic diversity

We hypothesized that the allopolyploid *A*. *suecica* would show larger phenotypic plasticity in changing environments than its parent species. Except for number of leaves at bolting in response to nutrients, this was not the case, and it rather seems that *A*. *arenosa* shows the greatest phenotypic plasticity (Figures 4 and 5; Table 3). As an allopolyploid species with multiple origins (Novikova et al., 2017) we asked if *A*. *suecica* had higher levels of genetic diversity than the parent species. However, we found that *A*. *arenosa* had the highest level of genetic diversity, probably due to its outcrossing nature. Both Lind‐Hallden et al. (2002) and Novikova et al. (2017) found *A*. *suecica* to possess the lowest genetic diversity among the three species. Novikova et al. (2017) also identified clear traces of genetic bottleneck and small number of founders for *A*. *suecica*, with overall levels of polymorphism lower than both *A*. *thaliana* and *A*. *arenosa* (30 and 12% lower, respectively) and a high number of non‐synonymous and putatively deleterious mutations. Meanwhile, our results indicate that some of the *A*. *thaliana* populations were the least genetically diverse among the three species, possibly due to lower levels of diversity in *A*. *thaliana* in this geographic region compared to other regions (Alonso‐Blanco et al., 2016).

### 4.3 The relationship between phenotypic plasticity, genetic diversity, and fitness

We identified a positive relationship between genetic diversity and phenotypic plasticity in response to environmental variation (Table 5). AFLP markers are often presumed to be neutral and mostly within non‐coding regions (Hufbauer, 2004), and thus high diversity in AFLP markers should not necessarily confer a higher expressional diversity. However, Caballero et al. (2013) investigated distribution of AFLP markers in the genome for several eukaryotic species. They found that for the *Eco*RI/*Mse*I system up to 87% of the markers were within coding regions, indicating that AFLP markers are not necessarily neutral. The positive relationship between phenotypic plasticity and genetic diversity might be explained by the species’ life histories. Both the larger plasticity, mainly as the result of more extreme responses to the light treatment, and higher genetic diversity could be due to *A*. *arenosa's* outcrossing, insect‐pollinated nature (Kilkenny & Galloway, 2008; Schoen & Brown, 1991).

It has been postulated that plasticity is favored if the environment is variable (Callaway et al., 2003; Charmantier et al., 2008; Lande, 2009; Valladares et al., 2014), and following this phenotypic plasticity could play an important role in a species’ ability to expand and adapt to novel environments (Davidson et al., 2011; Via et al., 1995). For instance, phenotypic plasticity increased a species’ tolerance to herbivore attacks (Agrawal, 2000). This may suggest that species with higher plasticity should show higher fitness, but it has proven difficult to establish a relationship between those two (Davidson et al., 2011; Hulme, 2008). Further on, fitness homeostasis (the ability to keep up reproduction when conditions get worse) is not necessarily favored by a high degree of plasticity in traits directly connected to fitness (Hulme, 2008). Even though *A*. *arenosa* show higher phenotypic plasticity and higher genetic diversity than *A*. *suecica* and *A*. *thaliana*, it does not show higher fitness as assessed by the *C* variable (Figure 6). When conditions get poor, *A*. *arenosa* show tendencies towards having the lowest fitness among the study species, illustrating the importance of separating between fitness and the ability to respond plastically to varying environmental conditions. In short‐lived species, it would not seem favorable to lower the number of flowers under poor conditions, as was observed in *A*. *arenosa*. Meanwhile, *A*. *thaliana* and *A*. *suecica* were more stable in number of flowers under various light conditions, which could imply a wider capacity for adapting to variable environments.

It should be noted that fitness is difficult to measure directly. Ideally, an experiment would run over several generations to quantify fitness, but this was not possible within the timeframe of this project. This study was restricted to measuring certain variables that could be considered more or less connected to fitness. A binary approach to this was chosen, classifying variables as either connected to fitness or not connected to fitness. The choice of variables connected to fitness in this experiment (flowers and biomass) was chosen based on methods used by Davidson et al. (2011). More flowers confer possibilities for higher offspring production. When it comes to biomass, Weiner et al. (2009) advocates an allometric relationship between biomass and reproduction. In that perspective, total biomass could be viewed as a good fitness proxy. Fitness is also a question of viability. Seed production and viability was not assessed in the experiment, but it should be included in future experiments.

## 5 CONCLUSIONS

In an environment that is changing faster than ever, understanding the mechanisms involved in range expansion and adaptation is important. Both phenotypic plasticity, genetic diversity, and polyploidy has been suggested as driving forces for introduction and adaptations to novel environments. This led us to investigate if the allopolyploid species *A*. *suecica* had more genetic diversity, a higher degree of phenotypic plasticity and a higher degree of fitness than its parent species *A*. *thaliana* and *A*. *arenosa*. We were unable to reveal any advantages for the allopolyploid *A*. *suecica* in our experiment in neither genetic diversity nor phenotypic plasticity. On the contrary, we found that *A*. *arenosa* had the highest level of diversity and phenotypic plasticity, probably due to its outcrossing nature. Across all species, we did find a positive relationship between phenotypic plasticity and genetic diversity, but this was not related to ploidy. *A*. *arenosa* showed tendencies towards having the lowest degree of fitness homeostasis, showing that there is no clear relationship between phenotypic plasticity and the ability to keep fitness high under varying conditions.

## AUTHORS' CONTRIBUTION

**Torbjørn Kornstad:** Conceptualization (equal); Formal analysis (lead); Investigation (lead); Methodology (lead); Visualization (lead); Writing – original draft (lead); Writing – review & editing (equal). **Siri Fjellheim:** Conceptualization (lead); Funding acquisition (lead); Project administration (lead); Resources (lead); Supervision (lead); Writing – original draft (supporting); Writing – review & editing (equal). **Mikael Ohlson:** Conceptualization (supporting); Supervision (supporting); Writing – original draft (supporting); Writing – review & editing (equal).

## CONFLICT OF INTEREST

The authors declare no competing interests.

## Data Availability

All data, that is, AFLP markers and phenotypic measurements, can be found in the DRYAD repository at https://doi.org/10.5061/dryad.dv41ns216.
